# A Diffusion-Based Detection Model for Accurate Soybean Disease Identification in Smart Agricultural Environments

**DOI:** 10.3390/plants14050675

**Published:** 2025-02-22

**Authors:** Jiaxin Yin, Weixia Li, Junhong Shen, Chaoyu Zhou, Siqi Li, Jingchao Suo, Jujing Yang, Ruiqi Jia, Chunli Lv

**Affiliations:** China Agricultural University, Beijing 100083, China

**Keywords:** soybean disease detection, endogenous diffusion sub-network, multi-task optimization, deep learning, precision agriculture

## Abstract

Accurate detection of soybean diseases is a critical component in achieving intelligent agricultural management. However, traditional methods often underperform in complex field scenarios. This paper proposes a diffusion-based object detection model that integrates the endogenous diffusion sub-network and the endogenous diffusion loss function to progressively optimize feature distributions, significantly enhancing detection performance for complex backgrounds and diverse disease regions. Experimental results demonstrate that the proposed method outperforms multiple baseline models, achieving a precision of 94%, recall of 90%, accuracy of 92%, and mAP@50 and mAP@75 of 92% and 91%, respectively, surpassing RetinaNet, DETR, YOLOv10, and DETR v2. In fine-grained disease detection, the model performs best on rust detection, with a precision of 96% and a recall of 93%. For more complex diseases such as bacterial blight and Fusarium head blight, precision and mAP exceed 90%. Compared to self-attention and CBAM, the proposed endogenous diffusion attention mechanism further improves feature extraction accuracy and robustness. This method demonstrates significant advantages in both theoretical innovation and practical application, providing critical technological support for intelligent soybean disease detection.

## 1. Introduction

Soybeans, as an essential crop, are not only a vital source of protein and oil but also widely used in food processing, livestock feed, and biopharmaceuticals [1,2,3]. In recent years, with the advancement of agricultural modernization, Bayannur has become a core area for premium soybean cultivation in China, significantly contributing to national food security and economic development. However, the outbreak of diseases during agricultural production poses a severe threat to soybean yield and quality [4]. Diseases can lead to direct yield losses and trigger issues such as soil degradation and continuous cropping obstacles [5]. With the expansion of cultivation areas and changing climatic conditions, the diversity of soybean diseases has increased, making traditional control measures less effective [6]. Hence, developing an efficient and accurate method for detecting soybean diseases is crucial for the sustainable development of Bayannur’s soybean industry. Such advancements not only improve agricultural productivity but also reduce pesticide use, fostering an environmentally friendly agricultural production model [7,8].

Currently, soybean disease detection primarily relies on the expertise of agricultural technicians. By visually observing lesion shape, color, and size, technicians can preliminarily identify disease types. However, this manual detection approach has significant limitations. First, it is inefficient and unsuitable for large-scale cultivation [9,10]. Second, the complexity of disease symptoms and the influence of environmental conditions, such as lighting and angles, make assessments highly subjective and uncertain. Moreover, the accuracy and consistency of detection results are often compromised by varying skill levels among technicians [11].

With the advancement of computer technology, traditional image-processing operators [12] and sliding window methods [13] have been applied to soybean disease detection. Traditional computer vision techniques extract features from images using predefined operators and locate diseased regions by implementing sliding window strategies. These methods focus on designing explicit feature descriptors to differentiate diseased areas from healthy regions. For example, SIFT (scale-invariant feature transform) [14,15] and HOG (histogram of oriented gradients) [16] are common feature extraction methods. SIFT detects keypoints in images and generates feature vectors that are invariant to scale and rotation [17]. While these methods demonstrate certain detection capabilities in laboratory settings, they rely heavily on handcrafted features and fixed window sizes, which limits their adaptability to diverse disease characteristics in real-world field environments. Challenges such as uneven lighting, complex backgrounds, and subtle symptoms significantly degrade detection performance. Additionally, the computational complexity of the sliding window approach makes it inefficient for processing large-scale images, further restricting its applicability in agricultural production.

In recent years, deep learning technology has achieved groundbreaking advancements in computer vision and has gradually been applied to agricultural disease detection [18,19]. Compared to traditional methods, deep learning models can automatically extract complex disease features from large datasets without manual feature design, offering higher robustness and generalization [20]. Convolutional neural networks (CNNs), as a representative of deep learning, have demonstrated exceptional performance in image classification, object detection, and semantic segmentation tasks. For instance, Tetila et al. utilized drone imagery and CNNs to detect and classify soybean pests, achieving a classification accuracy of 96% [21]. Razfar et al. developed a custom lightweight deep learning model to detect weeds in soybean crops, achieving a detection accuracy exceeding 95% [22]. Miao et al. proposed a sliding segmentation method based on CNNs for diagnosing bacterial leaf spot disease at different stages, achieving a recognition accuracy of 99.64% by optimizing parameters such as training epochs and batch size [23]. Annrose et al. constructed a cloud platform for soybean disease classification using a hybrid deep learning model optimized with the Archimedes algorithm, achieving a classification accuracy of 98% [24]. Bevers et al. applied transfer learning methods based on CNNs for disease identification from raw field images, achieving over 97% accuracy [25]. Similarly, Yu et al. proposed a residual neural network method based on transfer learning for recognizing soybean leaf diseases, achieving classification accuracies exceeding 98% [26]. However, these methods often face limitations in data diversity, image resolution, real-time performance, and adaptability to environmental conditions, affecting their generalization and practical effectiveness [21,27]. Despite the significant progress, challenges remain in applying deep learning models to agricultural scenarios [11].

Initially popular for image generation tasks, diffusion models have gained attention for their ability to model complex data distributions efficiently [28,29,30]. In the context of image detection, their key advantage lies in modeling high-dimensional features precisely, enabling effective capture of fine-grained details in diseased regions. In soybean disease detection, symptoms often exhibit multi-scale, nonlinear, and complex background characteristics. The progressive generation mechanism of diffusion models can adapt well to these complexities [31,32]. By integrating diffusion sub-networks with existing detection frameworks, these models significantly enhance the accuracy of disease localization and classification [33,34,35]. Data collection in agricultural contexts is challenging due to natural lighting, weather changes, and varying shooting angles, resulting in substantial sample variability and higher robustness requirements. Additionally, the uneven distribution of disease samples, where some disease types are underrepresented, makes models prone to overfitting.

Diffusion-based object detection models have recently gained significant attention for their ability to handle complex visual tasks, particularly in environments with high variability and noise [36]. These models are inspired by diffusion processes, commonly seen in physics, where they simulate the process of progressively adding noise to an image and then reversing it through a denoising process. This technique gradually refines the features of the image, which proves beneficial in handling intricate details in object detection tasks. A crucial innovation in diffusion-based object detection models is the introduction of an endogenous diffusion sub-network [37]. This sub-network improves feature distribution optimization by allowing the model to focus more effectively on the regions of interest, such as disease spots in agricultural images. By injecting noise into the feature maps and then denoising them step by step, the model learns to retain important object details while suppressing background noise, which is particularly useful in complex scenes where object boundaries are unclear [38]. This process allows the model to better identify and classify objects, even in the presence of complex backgrounds or when objects appear as small or partially occluded. Another advantage of diffusion models over traditional object detection approaches, such as anchor-based models like RetinaNet or YOLO, is the flexibility in feature extraction [33]. Traditional methods rely on predefined anchor boxes to detect objects, which often struggle with detecting irregularly shaped objects or objects with varied appearances. Diffusion-based models, on the other hand, do not rely on fixed anchor boxes, instead modeling features progressively. This enables them to adapt to more diverse and complex scenarios, such as varying object shapes and sizes or more difficult environmental conditions like varying lighting or cluttered backgrounds [39]. One of the distinguishing characteristics of diffusion-based models compared to Transformer-based methods like DETR and DETR v2 is the ability to avoid the computational complexity of global self-attention mechanisms while still maintaining the model’s ability to process complex relationships within the data. While Transformer models excel at capturing long-range dependencies in the input data, their computational overhead can be prohibitive, particularly for high-resolution images or real-time applications [40]. Diffusion-based models alleviate this issue by incrementally improving feature maps, which makes them more efficient in extracting relevant features without requiring the full-scale computational cost of self-attention.

To address these challenges, this study leverages the powerful characteristics of diffusion models to propose a diffusion-based deep learning method for the efficient detection of soybean diseases in Bayannur. This study focuses on the practical needs of soybean disease detection in Bayannur and proposes an innovative detection network based on diffusion models, with the following key contributions:

1. Introduction of a diffusion detection network: Leveraging the strong feature modeling capabilities of diffusion models in generative tasks, a diffusion sub-network is designed to capture fine-grained features of diseased areas.
2. Novel internal diffusion loss function: An internal diffusion loss function optimized for diseased areas is proposed, assigning adaptive weights to different disease categories to enhance classification and localization accuracy.
3. Data augmentation and robustness improvement: Diverse data augmentation techniques, are utilized to expand the dataset and improve model adaptability to complex field environments.
4. Model deployment optimization: The model is optimized for inference speed and resource usage to meet the practical application needs of Bayannur’s fields, enabling efficient edge-device deployment.

In summary, the proposed method not only surpasses traditional models in detection performance but also demonstrates high practicality, providing robust support for the intelligent and sustainable development of Bayannur’s soybean industry.

## 2. Materials and Methods

### 2.1. Dataset Collection

The collection of the dataset is a crucial step in the development of soybean disease detection models, as it directly impacts the effectiveness of model training and the accuracy of the final detection results. In this study, we collected image data from two primary sources: the Bayan Nur Forestry Center, which provides images captured in real field conditions, and publicly available online image resources, which offer additional diversity in disease types. These images cover the most common types of soybean diseases, including rust, root rot, powdery mildew, Fusarium head blight, and bacterial blight. Each disease is represented by a sufficient number of images, ranging from 1800 to 8000, ensuring the dataset’s diversity and providing a robust sample size for effective model training and validation. This diversity includes variations in disease stages, severity, and environmental conditions, which enhances the model’s ability to generalize across different real-world scenarios. The specific image counts for each disease are shown in Table 1.

During the collection process at the Bayan Nur Forestry Center, we utilized high-resolution digital cameras and specialized close-up photography equipment equipped with Canon EOS 5D Mark IV cameras (Beijing, China) and Canon EF 100 mm f/2.8 L Macro IS USM macro lenses (Beijing, China), paired with Canon MR-14EX II Ring Lite ring flashlights (Beijing, China). These setups ensured clear disease images even under suboptimal lighting conditions. Additionally, to simulate real-world field observation conditions, images were captured at different times of the day, including morning, noon, and evening. This approach allowed us to collect disease images under varying natural lighting conditions, further enhancing the dataset’s generalizability. The collection of online images was conducted using search engines and agricultural research databases, focusing on publicly available resources that permit academic use. These images, often uploaded by other research institutions or agricultural experts, encompass a variety of scenarios ranging from laboratory settings to real field conditions. Although online images are less controlled in terms of conditions, they provide a broader representation of disease manifestations and more diverse backgrounds, helping the model learn more complex disease characteristics.

**Figure 1:**
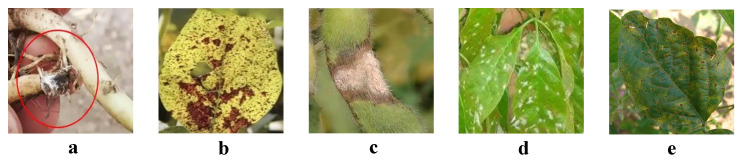
Dataset samples: (**a**) is powdery mildew; (**b**) is rust; (**c**) is root rot; (**d**) is Fusarium head blight; (**e**) is bacterial blight.

At every step of the data collection process, detailed records were kept for each image, including the specific time, location, camera settings (e.g., aperture, shutter speed, and ISO), and the specific location and manifestations of the disease in the image. For example, shutter speed was typically set at 1/200 s, ISO sensitivity between 400 and 800, and aperture at f/5.6 to f/8 to maintain clarity and minimize noise in varying lighting conditions. For each disease, we also documented its typical symptoms, as shown in Figure 1. For example, rust is characterized by small yellow or reddish-brown spots on leaves, which expand and form rust-like powder as the disease progresses. Root rot appears as black or brown rot on the roots of soybeans, often leading to overall wilting and death of the plant. Powdery mildew manifests as a white, powdery coating on the surface of leaves, eventually covering the entire leaf area over time. Fusarium head blight primarily affects the stems of plants, causing red or purple streaks and often softening the infected areas. Bacterial blight is characterized by dark green or yellow water-soaked spots on leaves, which often merge into larger lesions.

This comprehensive data collection and meticulous recording process not only provided high-quality input data for the development and testing of the soybean disease detection model but also contributed valuable data resources for future agricultural disease research.

### 2.2. Data Augmentation

**Figure 2:**
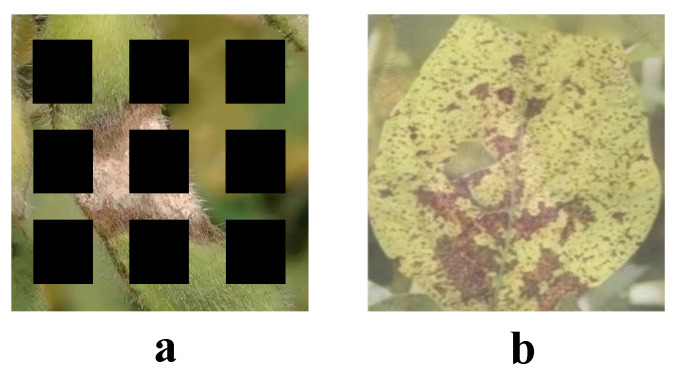
Different image enhancement methods: (**a**) is GridMask; (**b**) is Mixup.

Data augmentation is crucial for enhancing deep learning model performance, especially in agricultural tasks like soybean disease detection, where datasets are often limited and imbalanced. This study applied both classic methods, such as flipping, rotation, and cropping, and novel techniques like GridMask and Manifold Mixup to diversify the dataset and improve model robustness and generalization, as shown in Figure 2. The data augmentation is applied before model training, and the augmentation operations are applied to each training image. To prevent the model from overfitting to the original data during training, data augmentation diversifies the training set by generating more transformed image samples, allowing the model to learn a wider range of features. These augmentation operations are automatically executed through an image preprocessing pipeline before training and do not affect the test or validation sets, ensuring the fairness of evaluation results.

#### 2.2.1. Classic Augmentation

In classic data augmentation methods, flipping, rotation, and cropping are the most commonly used operations. In the flipping operation, we apply horizontal flipping to each image, which means performing a left-right mirror flip of the input image. This operation helps the model learn stronger spatial invariance. The probability of applying the flip is set to 50%, meaning that each training image has a 50% chance of being horizontally flipped. This proportion ensures the diversity of the augmentation while avoiding the negative effects of excessive augmentation. The rotation operation involves randomly rotating the image by different angles to enhance the model’s adaptability to different perspectives and orientations. We set the rotation angle range from −30 to +30 degrees, with the rotation operation also being applied to each image with a 50% probability. Through random rotations, the model can learn the features of disease regions at different rotation angles and cope with the challenges posed by various shooting angles. The cropping operation is designed to enhance the model’s ability to perceive different regions of the image by randomly cropping areas of the original image, increasing the diversity of local features. Specifically, we randomly crop a region of the image with a crop ratio ranging from 0.8 to 1 of the original image. After cropping, the cropped image is resized to the original size to ensure consistency in input image dimensions. The purpose of this operation is to highlight the disease features in different regions through cropping, preventing the model from relying too much on the global background information, and thereby improving the recognition ability of local details.

#### 2.2.2. GridMask

GridMask augments data by overlaying regularly distributed occlusion grids on images, simulating scenarios where data are partially missing. The grid’s position, size, and shape can be randomized to generate diverse training samples. The augmentation process can be expressed as follows [41]:

(1) $$\tilde{I}\left(x,y\right)=I\left(x,y\right)\cdot M\left(x,y\right)$$

where $\tilde{I}\left(x,y\right)$ represents the augmented image, and M(x,y) is the occlusion grid mask. The mask value is 1 to retain the pixel value or 0 to occlude the pixel. The mask M(x,y) is generated based on grid parameters, such as spacing and cell size. By simulating scenarios with partial data loss, GridMask enhances the model’s ability to adapt to incomplete information, contributing to improved robustness in detection tasks.

#### 2.2.3. Manifold Mixup

Unlike image-space augmentation methods, Manifold Mixup operates within the hidden feature space of deep learning models. Specifically, Manifold Mixup performs linear interpolation on feature vectors in the hidden layers to generate new sample features and corresponding labels. Given hidden layer features $z_{A}$ and $z_{B}$ with their respective labels $y_{A}$ and $y_{B}$, the mixing process is defined as [42]:

(2) $$\tilde{z}=\lambda z_{A}+\left(1-\lambda \right)z_{B}$$

(3) $$\tilde{y}=\lambda y_{A}+\left(1-\lambda \right)y_{B}$$

where λ is a randomly sampled weight coefficient, typically drawn from a Beta(α,α) distribution. By performing data augmentation in the feature space, Manifold Mixup enables the model to learn higher-dimensional semantic information, thereby improving its generalization capability.

### 2.3. Proposed Method

**Figure 3:**
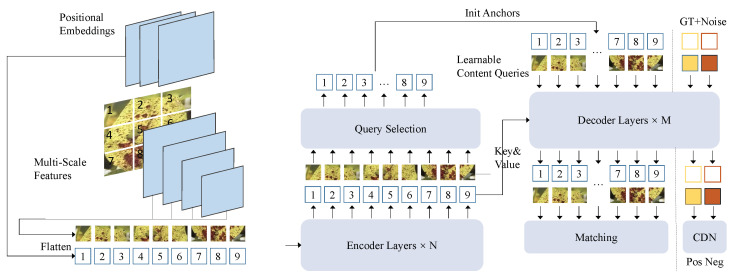
Overview of the proposed method.

This paper proposes a soybean disease detection method based on the diffusion mechanism and an object detection framework, as shown in Figure 3. The core model structure includes three main modules: feature extraction module, endogenous diffusion sub-network, and object detection module. Processed soybean disease image data are sequentially passed through these three modules, completing the full workflow from feature extraction to the localization and classification of disease regions. The following sections describe the functionality of each module and their interconnections in detail.

The feature extraction module forms the foundation of the entire network, with the primary function of converting input soybean disease images into multi-scale feature representations. This module uses an improved convolutional neural network (e.g., ResNet-50) and extracts local and global features from the images through stacked convolutional layers, pooling layers, and skip connections to generate multi-scale feature maps $F_{multi}$. The endogenous diffusion sub-network is the core module of the proposed method, designed to further enhance the semantic representation and fine-grained modeling of features through the diffusion mechanism. The object detection module is based on an improved Transformer architecture, consisting of multiple decoder layers and matching mechanisms. Its main function is to perform candidate box generation, classification, and localization on the feature maps $F_{fusion}$ generated by the endogenous diffusion sub-network.

#### 2.3.1. Diffusion-Detection Network Model

**Figure 4:**
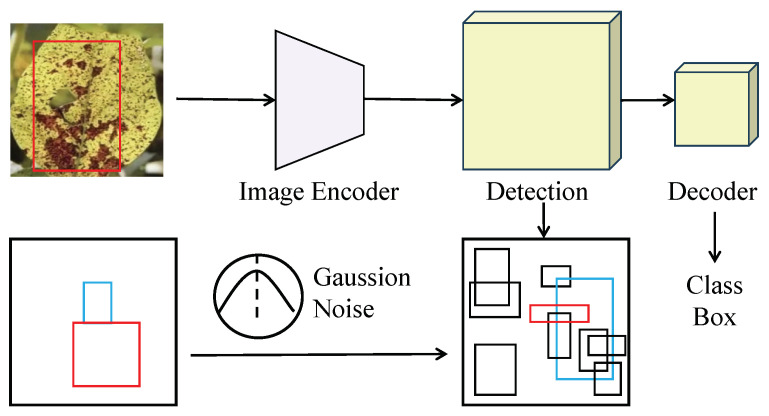
Flowchart of the diffusion-detection network model.

This work presents a diffusion-detection network model, a deep learning framework specifically designed for soybean disease detection tasks, as shown in Figure 4. The model integrates diffusion mechanisms with object detection networks to achieve high-precision and robust localization and classification of disease regions. The entire network consists of three tightly connected modules: the feature extraction module, the endogenous diffusion sub-network, and the object detection module. Together, they form a complete pipeline, from feature extraction to object recognition. The model design leverages the progressive feature modeling capabilities of the diffusion mechanism while incorporating multi-task optimization strategies from the object detection framework, enhancing detection accuracy and adaptability to complex scenarios.

**Figure 5:**
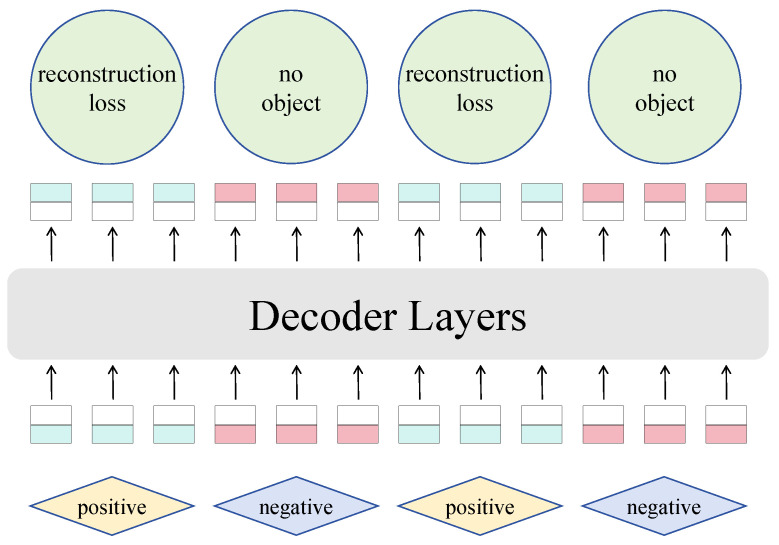
Architecture of the decoder.

The input to the diffusion-detection network is preprocessed images of diseased soybeans, which are first passed through the feature extraction module to generate multi-scale feature maps. The feature extraction module employs a lightweight convolutional neural network (e.g., ResNet-50) as the backbone. This module processes the input images with multiple convolutional layers, extracting multi-scale feature representations. The output feature maps have dimensions H×W×C, where *H* and *W* denote the height and width, and C=256 represents the number of channels. These feature maps serve as input for both the endogenous diffusion sub-network and the object detection module. The endogenous diffusion sub-network, the core module of the model, performs progressive modeling and optimization of the input feature maps through a diffusion mechanism. The forward diffusion module gradually adds Gaussian noise to the input feature maps, generating a sequence of perturbed feature maps ${\left\{F_{t}\right\}}_{t=1}^{T}$, where *T* denotes the number of diffusion steps. The mathematical formulation of forward diffusion is as follows:

(4) $$F_{t}=\sqrt{1-\beta_{t}}F_{t-1}+\sqrt{\beta_{t}}\epsilon ,\;\epsilon ∼N\left(0,I\right),$$

where $F_{0}=F_{multi}$ represents the input feature map, $\beta_{t}$ is the linearly increasing noise intensity, and ϵ is standard Gaussian noise. The primary objective of forward diffusion is to simulate feature perturbations under complex conditions, such as lighting variations and background noise, thereby improving the model’s robustness and generalization capability. The reverse generation module progressively denoises the perturbed feature maps, restoring them to feature representations close to the target distribution and ultimately generating optimized feature maps $F_{0}^{\prime }$. The mathematical formulation of reverse generation is as follows:

(5) $$F_{t-1}=\mu_{\theta }\left(F_{t},t\right)+\Sigma_{\theta }\left(F_{t},t\right)\epsilon ,$$

where $\mu_{\theta }\left(F_{t},t\right)$ and $\Sigma_{\theta }\left(F_{t},t\right)$ are parameterized mean and covariance functions, and ϵ∼N(0,I) is standard Gaussian noise. The reverse generation module is implemented with multi-layer convolutional networks, where each layer has a kernel size of 3×3 and C=256 channels. ReLU activation functions and batch normalization are applied to ensure smooth and stable feature transformations. The sequence of denoised feature maps generated by the reverse generation module is fused into the final feature representation using a weighted summation:

(6) $$F_{fusion}=\sum_{t=1}^{T}w_{t}F_{t},\;w_{t}=\frac{\exp(-\beta_{t})}{\sum_{k=1}^{T}\exp\left(-\beta_{k}\right)},$$

where $w_{t}$ are dynamic weights based on the diffusion step, balancing the contributions of different diffusion steps to the final feature optimization. The object detection module uses a Transformer-based decoder structure to perform classification and bounding box regression on candidate regions, as shown in Figure 5.

The decoder takes the fused feature map $F_{fusion}$ generated by the endogenous diffusion sub-network as input, along with a set of learnable query vectors. Through multi-layer self-attention mechanisms, it predicts target categories and bounding box positions. The output of the object detection module includes the classification confidence and precise bounding box parameters for each candidate region. The classification and regression tasks are optimized using cross-entropy loss and Smooth L1 loss, respectively. The diffusion-detection network is fundamentally designed to enhance the progressive modeling of features through the diffusion mechanism, enabling better capture of fine-grained details in disease regions under complex scenarios. Unlike traditional object detection networks that rely on global self-attention mechanisms, this model achieves significant improvements in feature representation and task adaptability through the collaborative optimization of forward diffusion and reverse generation. Mathematically, the reconstruction constraint imposed on the diffusion-generated features ensures that the final feature distribution aligns with the original distribution:

(7) $$E\left[F_{0}^{\prime }\right]=E\left[F_{multi}\right],\;\mathrm{as}\;t\to 0.$$

This property validates that the diffusion mechanism effectively preserves the semantic consistency of the features under noise perturbation while enhancing sensitivity to target regions. In soybean disease detection tasks, the diffusion-detection network adapts effectively to complex field conditions through its progressive feature modeling approach, improving detection precision and robustness, and providing vital support for the development of precision agriculture.

#### 2.3.2. Endogenous Diffusion Sub-Network

**Figure 6:**
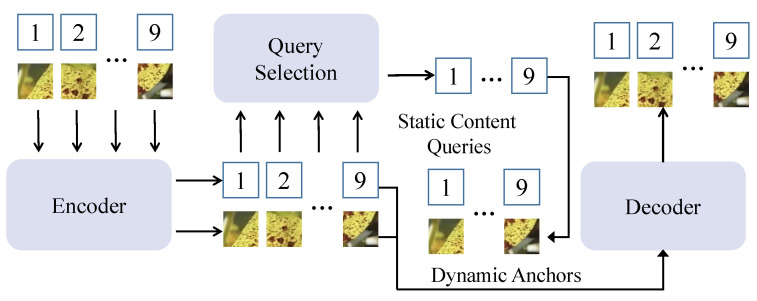
Architecture of endogenous diffusion sub-networks.

The endogenous diffusion sub-network is the core module of the proposed diffusion-detection network model, as shown in Figure 6. Its design is based on the progressive feature generation mechanism of the diffusion model. Through forward diffusion and reverse generation, the sub-network enables feature modeling and optimization. Compared to traditional object detection frameworks such as the Deformable Transformer for end-to-end object detection (DETR)’s self-attention mechanism, the endogenous diffusion sub-network introduces progressive perturbation and denoising into the feature extraction process, allowing features to better adapt to complex scenarios (e.g., variations in field lighting and background interference) while effectively capturing fine-grained details of disease regions.

The input to the endogenous diffusion sub-network is the multi-scale feature map $F_{multi}$ generated by the feature extraction module, with dimensions H×W×C, where *H* and *W* denote the height and width, and C=256 represents the number of channels. This network consists of a forward diffusion module and a reverse generation module, which progressively models and optimizes the input features. The forward diffusion module injects noise into the feature map, generating a sequence of feature maps with varying levels of perturbation ${\left\{F_{t}\right\}}_{t=1}^{T}$, where *T* is the number of diffusion steps. The purpose of forward diffusion is to simulate environmental complexities such as lighting changes and background noise by injecting noise, thereby improving the robustness of the features. In this study, the number of diffusion steps *T* is set to 100, and $\beta_{t}$ ranges from 0.0001 to 0.02, ensuring gradual noise perturbation and stable feature distribution. The reverse generation module progressively denoises the noisy feature maps, restoring them to feature representations close to the target distribution, and ultimately generates the optimized feature map $F_{0}^{\prime }$. Implementation-wise, the reverse generation module is composed of three convolutional layers, each with a kernel size of 3×3 and C=256 channels. The activation function used is ReLU, and Batch Normalization is applied to ensure feature regularization. The output of the reverse generation module is a sequence of denoised feature maps ${\left\{F_{t}\right\}}_{t=1}^{T}$, with the final feature map $F_{0}^{\prime }$ combining the results of multiple denoising steps and encapsulating rich fine-grained feature information.

Unlike DETR’s traditional self-attention mechanism, the endogenous diffusion sub-network does not rely on global self-attention to directly model features. Instead, it enhances feature representations through the progressive modeling approach of the diffusion mechanism. While self-attention mechanisms focus on capturing long-range dependencies between features, they may underperform in scenarios involving small-object detection or significant noise interference. In contrast, the endogenous diffusion sub-network improves feature robustness through forward diffusion, captures fine-grained details of disease regions through reverse generation, and preserves global semantic information through weighted fusion. This progressive modeling design not only enhances the detection of small disease targets but also demonstrates excellent robustness in complex field scenarios.

This property guarantees that the model retains the semantic consistency of features under noise perturbation while improving sensitivity to target regions. In the context of soybean disease detection, this module effectively extracts disease region features under varying lighting conditions, complex backgrounds, and imbalanced disease sample distributions. These characteristics enable the endogenous diffusion sub-network to achieve outstanding performance in field scenarios, providing strong support for the precision and robustness of soybean disease detection.

#### 2.3.3. Endogenous Diffusion Loss Function

In the proposed diffusion-detection network model, an adaptive endogenous diffusion loss function is designed to optimize feature learning and detection performance. This loss function integrates the feature reconstruction objectives of the diffusion model with the classification and localization requirements of the object detection task. Compared to traditional independent loss designs, such as cross-entropy loss and Smooth L1 loss, the endogenous diffusion loss function incorporates supervisory signals into the diffusion generation process, achieving a deep coupling of feature modeling and task optimization. This approach effectively enhances robustness and detection accuracy under complex scenarios. The overall form of the loss function is as follows:

(8) $$L_{\mathrm{diffusion}}=\sum_{t=1}^{T}\alpha_{t}L_{\mathrm{reconstruction}}\left(F_{t},F_{multi}\right)+L_{\mathrm{classification}}+L_{\mathrm{regression}},$$

where $L_{\mathrm{reconstruction}}$ represents the feature reconstruction loss, which enforces the progressive approximation of the diffusion-generated features to the original feature distribution. $L_{\mathrm{classification}}$ and $L_{\mathrm{regression}}$ correspond to the classification and regression tasks, respectively. $\alpha_{t}$ is a weight associated with the time step *t*, dynamically adjusting the contribution of different diffusion steps to the overall loss. In this design, $\alpha_{t}$ is set as a decreasing weight, indicating that diffusion steps closer to the original feature distribution contribute more significantly to the final loss. Feature reconstruction loss $L_{\mathrm{reconstruction}}$ is the core component of the endogenous diffusion loss function. By constraining the Euclidean distance between the diffusion process features $F_{t}$ and the original features $F_{multi}$, the model maintains the semantic consistency of the feature map while undergoing noise perturbations. Its formulation is as follows:

(9) $$L_{\mathrm{reconstruction}}\left(F_{t},F_{multi}\right)=\frac{1}{N}\sum_{i=1}^{N}{∥F_{t}^{\left(i\right)}-F_{multi}^{\left(i\right)}∥}_{2}^{2},$$

where *N* denotes the total number of pixels in the feature map, and $F_{t}^{\left(i\right)}$ and $F_{multi}^{\left(i\right)}$ represent the feature values at pixel *i* in the diffusion and original feature maps, respectively. Through the iterative optimization of this reconstruction loss, the diffusion-generated features progressively approximate the original feature distribution, enhancing robustness to lighting changes and noise interference. Classification loss $L_{\mathrm{classification}}$ adopts focal loss to address the class imbalance in the disease categories. Focal loss assigns lower weights to easily classified samples, emphasizing the impact of harder-to-classify samples, thus improving the detection performance for underrepresented disease classes. Its formulation is as follows:

(10) $$L_{\mathrm{classification}}=-\frac{1}{M}\sum_{j=1}^{M}\omega_{j}\cdot {\left(1-p_{j}\right)}^{\gamma }\log\left(p_{j}\right),$$

where *M* is the total number of samples, $p_{j}$ is the predicted probability of the *j*-th sample belonging to the target class, $\omega_{j}$ is the class weight, and γ is a modulating factor (set to 2 in this work) to control the emphasis on harder-to-classify samples. Regression loss $L_{\mathrm{regression}}$ employs Smooth L1 loss to optimize the prediction of bounding boxes for disease regions. Compared to standard L1 loss, Smooth L1 loss offers smoother gradient behavior for small errors, accelerating model convergence while improving robustness to outliers. Its formulation is as follows:

(11) $$L_{\mathrm{regression}}=\frac{1}{K}\sum_{k=1}^{K}\mathrm{SmoothL}1\left(b_{k},{\hat{b}}_{k}\right),$$

where *K* is the total number of predicted bounding boxes, $b_{k}$ is the predicted bounding box, and ${\hat{b}}_{k}$ is the ground truth bounding box. Smooth L1 loss is defined as follows:

(12) $$\mathrm{SmoothL}1\left(x\right)=\left\{\begin{array}{cc} 0.5x^{2}, & \mathrm{if}\;|x|<1,\\ |x|-0.5, & \mathrm{otherwise}. \end{array}\right.$$

The primary motivation for designing the endogenous diffusion loss function is to combine feature learning during the diffusion process with the task optimization of object detection, forming a closed-loop optimization system. On the feature modeling level, $L_{\mathrm{reconstruction}}$ enhances the semantic consistency between the diffusion-generated and original features. On the task optimization level, $L_{\mathrm{classification}}$ and $L_{\mathrm{regression}}$ refine the classification and localization accuracy for disease regions, enabling joint optimization across multiple tasks.

This property ensures the effectiveness of the progressive denoising process during reverse generation. Additionally, the stepwise optimization strategy in the endogenous diffusion loss function dynamically adjusts the weights $\alpha_{t}$ for different diffusion steps, effectively balancing the trade-off between feature modeling and task optimization. This ensures that the final feature map achieves optimal performance in both fine-grained details and global semantic representation. Compared to traditional loss functions, the endogenous diffusion loss function offers significant advantages. First, the introduction of feature reconstruction loss enables diffusion-generated features to better adapt to complex scenarios, such as field lighting variations and background noise, significantly enhancing model robustness. Second, classification loss through the focal mechanism addresses class imbalance issues, substantially improving detection performance for rare disease categories. Finally, the deep integration of task optimization and feature modeling results in superior accuracy and stability in classification and localization tasks. This design ensures that the endogenous diffusion loss function supports the diffusion-detection model in achieving high precision and robustness in soybean disease detection under complex real-world conditions.

### 2.4. Experimental Setup

#### 2.4.1. Hardware Platform

The hardware setup included a workstation equipped with a high-performance Graphics Processing Unit (GPU), specifically an NVIDIA A100 (80 GB VRAM), known for its exceptional computational capabilities in deep learning tasks. Additionally, the Central Processing Unit (CPU) was an Intel Xeon Gold 6226R processor with 24 cores and a base clock speed of 2.9 GHz, providing efficient support for data preprocessing and task scheduling. To handle large-scale data processing and model storage, the system was equipped with a 2 TB NVMe SSD and 256 GB DDR4 RAM, ensuring stability during data loading and training. The hardware platform was designed with a balance of high performance and scalability, offering robust support for complex deep learning tasks.

#### 2.4.2. Software Platform

On the software side, the experiments were developed and executed on Ubuntu 20.04, chosen for its stability and broad compatibility with deep learning frameworks. The deep learning framework used was PyTorch 2.0, renowned for its flexible dynamic computation graph and efficient GPU acceleration, meeting the demands of diffusion models and object detection models. Data augmentation was performed using the Albumentations library, valued for its extensive set of augmentation operators and support for custom strategies. CUDA 12.0 and its associated cuDNN library were utilized to fully leverage GPU parallel computing capabilities during model training and evaluation. TensorBoard and Matplotlib were employed to document the experimental process and visualize results, while hyperparameter optimization and result analysis were conducted using Optuna and Pandas. This combination of software tools not only accelerated the experiment development process but also significantly enhanced the reliability and flexibility of model training.

#### 2.4.3. Dataset Splitting and Hyperparameter Configuration

To comprehensively evaluate the model’s performance, the collected soybean disease dataset was divided into training, validation, and test sets at a ratio of 70%, 15%, and 15%, respectively. This split ensured sufficient data for model training while preserving adequate amounts for validation and testing, providing stable and reliable evaluation results. For hyperparameter settings, a combination of empirical and experimental optimization approaches was adopted. The initial learning rate (α) was set to 0.001 and adjusted dynamically using a cosine annealing schedule to accelerate convergence during training. The AdamW optimizer was used, with a weight decay coefficient set to 0.01 to balance model generalization and overfitting risk. The batch size was set to 32 to maximize GPU memory utilization while maintaining gradient stability. In this study, the model training process was designed for 100 epochs, with early stopping based on changes in validation performance to avoid ineffective training and overfitting. Specifically, during training, the convergence of the model was assessed by monitoring the training loss and validation loss. The training loss curve typically decreases gradually with the increase in epochs, until it converges and stabilizes, while the validation loss is used to determine if overfitting occurs. If the validation loss does not significantly decrease over several epochs, it indicates that the model has reached an optimal state. Consequently, early stopping was triggered to halt further training, ensuring that the model converges to the best performance in fewer training iterations.

Regarding the selection of 100 epochs, multiple tests were conducted during the experiments, which revealed that both the training loss and validation loss plateaued after 100 epochs, with no further improvement in performance or signs of overfitting on the validation set. Therefore, 100 epochs were chosen as the maximum training duration, ensuring that the model effectively learned the features of the data while avoiding the computational overhead and overfitting issues associated with excessive training. For the hyperparameter settings in the diffusion model, 100 diffusion steps were used during training. This setting ensured that the diffusion process could gradually optimize feature distribution, simulating noise and interference in complex scenarios. The noise intensity ($\beta_{t}$) values increased linearly from the minimum value of $\beta_{\min}=0.0001$ to the maximum value of $\beta_{\max}=0.02$. This design allowed the noise to gradually intensify, helping the model learn complex backgrounds and fine-grained features while maintaining stability. It is important to note that $\beta_{\min}$ and $\beta_{\max}$ represent the minimum and maximum noise intensities during the diffusion process, and their linear increase helps to perturb the features progressively while avoiding excessive noise interference. In the object detection network, the non-maximum suppression (NMS) threshold was set to 0.5 to filter out redundant detection boxes, and a confidence threshold of 0.3 was set to reduce interference from low-confidence predictions. These settings further improved the detection accuracy and robustness. The training loss curve illustrates the process of the model gradually learning features and reaching a stable state, while the validation loss curve shows the performance change on the validation set, providing the basis for triggering the early stopping mechanism.

#### 2.4.4. Baseline

In the soybean disease detection task, several mainstream object detection models were selected as baseline models for comparative experiments to comprehensively evaluate the performance of the proposed method. These include RetinaNet, YOLOv10, DETR, and DETR v2, representing different stages of development and technological directions in the field of object detection. RetinaNet is a classic single-stage detector [43] that significantly improves the detection of small objects and challenging samples with its proposed focal loss. The core idea is to address class imbalance by reducing the weight of easily classified samples. YOLOv10, as the latest version of the YOLO series, further optimizes the feature extraction network and multi-scale detection strategies, achieving higher accuracy while maintaining real-time detection speed [44]. DETR introduced the groundbreaking Transformer architecture, leveraging the self-attention mechanism to globally model relationships between objects. This eliminates the need for traditional post-processing steps such as NMS [45]. DETR v2 builds on this foundation, further enhancing performance through more efficient feature extraction modules and improved optimization strategies [46].

These baseline models span a range of paradigms, from single-stage to two-stage detectors and from convolutional network-based approaches to Transformer-based architectures, providing a multidimensional performance comparison for the proposed method. Through experiments, the proposed diffusion model’s innovation and advantages in soybean disease detection can be quantified, particularly in terms of improvements in accuracy, recall, and robustness under complex scenarios.

### 2.5. Evaluation Metrics

The choice of evaluation metrics is critical for comprehensively assessing the model’s performance in classification and object detection tasks. This study employed a set of commonly used metrics, including accuracy, precision, recall, and mean average precision (mAP). These metrics evaluate the model’s ability to classify and detect diseases from different perspectives, measuring overall sample recognition accuracy as well as performance across different classes, especially in cases of imbalanced disease sample distributions. Furthermore, mAP@50 and mAP@75, corresponding to Intersection over Union (IoU) thresholds of 0.5 and 0.75, were used to explore the model’s accuracy and stability in detection tasks under varying stringency.

Accuracy measures the overall correct classification rate across all samples and is defined as [47]:

(13) $$Accuracy=\frac{TP+TN}{TP+TN+FP+FN}$$

where TP (true positive) denotes the number of correctly classified positive samples, TN (true negative) denotes correctly classified negative samples, FP (false positive) represents negative samples misclassified as positive, and FN (false negative) represents positive samples misclassified as negative. While intuitive, accuracy may lose representativeness in cases of class imbalance, necessitating complementary metrics for a more comprehensive analysis.

Precision evaluates the accuracy of positive predictions and is particularly relevant in object detection tasks. It is defined as follows [48]:

(14) $$Precision=\frac{TP}{TP+FP}$$

Recall measures the model’s ability to capture all positive samples and is defined as follows:

(15) $$Recall=\frac{TP}{TP+FN}$$

mAP is the most important evaluation metric in object detection, balancing precision and recall. It is calculated based on the precision–recall (P(R)) curve, which plots precision versus recall at various prediction thresholds. The average precision (AP) for a single class is defined as the area under the PR curve:

(16) $$AP=\int_{0}^{1}P\left(R\right)\;dR$$

and the mAP for multiple classes is the mean of APs across all classes [49]:

(17) $$mAP=\frac{1}{C}\sum_{c=1}^{C}AP_{c}$$

where *C* is the total number of classes, and AP_c is the average precision for class *c*. Specifically, mAP@50 refers to the average precision at an IoU threshold of 0.5, while mAP@75 corresponds to an IoU threshold of 0.75. These metrics capture the model’s performance under varying levels of detection strictness.

By combining these metrics, the model’s classification and detection performance in soybean disease detection can be comprehensively evaluated, providing reliable insights for model optimization and real-world deployment. All metrics were meticulously recorded during the experiments, and 5-fold cross-validation was employed to further validate their stability and generalization, offering robust support for advancing intelligent soybean disease detection.

## 3. Results and Discussion

### 3.1. Disease Detection Experiment Results

**Figure 7:**
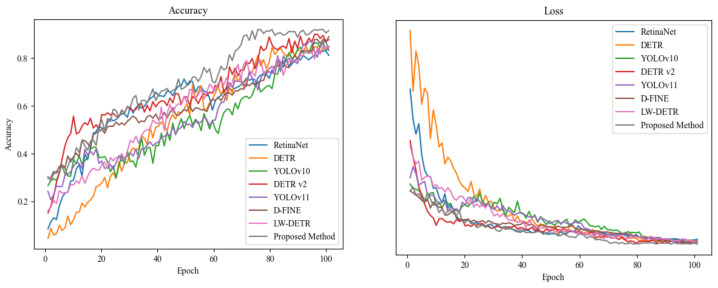
Training process.

The purpose of this experiment is to compare the performances of different object detection models in disease detection tasks and to verify the effectiveness and advantages of the proposed diffusion-based object detection model. The experiment includes comparisons with several classic and emerging object detection models, such as RetinaNet [43], DETR [50], YOLOv10 [44], DETR v2 [51], YOLOv11 [52], D-FINE [53], and LW-DETR [54]. By evaluating the performances of these models based on metrics such as precision, recall, accuracy, and mean average precision (mAP@50, mAP@75, mAP50-95), the aim is to quantify the performance differences among models when applied to soybean disease detection tasks and provide theoretical support for improvements to the proposed method. The experimental results are shown in Table 2 and Figure 7.

Firstly, for RetinaNet, it is explicitly stated that ResNet-50 is used as the backbone network, and the model is optimized with Focal Loss. Additionally, ImageNet pre-trained weights are utilized, providing the model with a robust initial feature representation, which aids in faster convergence and enhances detection accuracy. For DETR, it is clearly mentioned that a Transformer-based architecture is employed, with ResNet-50 serving as the backbone, and the model also utilizes ImageNet pre-trained weights to leverage features learned from large-scale datasets. YOLOv10 adopts an improved YOLO architecture, enhancing its detection capability by introducing multi-scale feature maps. CSPDarknet is used as the backbone network, and ImageNet pre-trained weights are again used to accelerate training and improve model performance. Finally, for DETR v2, it is noted that optimizations have been made based on DETR, including the use of more efficient feature extraction modules, while still using ResNet-50 as the backbone network, along with ImageNet pre-trained weights. By including these detailed pieces of information in the table, the aim is to provide readers with a more comprehensive understanding of the design of the different models and to highlight the potential impact of these factors on the experimental results. The results show that RetinaNet performs relatively poorly across all metrics, with a precision of 0.85, recall of 0.80, and mAP@50 of 0.82. This can be attributed to RetinaNet’s reliance on a single-stage detection framework, which has limited ability to handle small targets and complex backgrounds in disease regions [55]. DETR demonstrates improvements over RetinaNet in most metrics, with a precision of 0.87, recall of 0.83, and mAP@50 of 0.85. This is due to DETR’s Transformer-based global modeling capability, which effectively captures long-range dependencies in disease regions [56]. However, DETR’s slow convergence and lack of progressive fine-grained feature modeling in high-resolution images highlight areas for further improvement [57]. YOLOv10 outperforms both RetinaNet and DETR, achieving a precision of 0.88, recall of 0.85, and mAP@50 of 0.88. This is due to its optimized feature extraction network and multi-scale detection strategy, which balance real-time processing and detection accuracy [58]. DETR v2 further enhances performance by introducing more efficient feature extraction modules and improved optimization strategies, achieving a precision of 0.90, recall of 0.87, and mAP@50 of 0.90, demonstrating robustness under complex backgrounds and varying lighting conditions [46]. Among the emerging object detection models, YOLOv11 demonstrates strong detection capabilities, with a precision of 0.89, recall of 0.86, and mAP@50 of 0.87. Its performance is close to that of DETR v2, showing the advantages of the YOLO series in real-time and high-precision detection. D-FINE enhances the robustness of the model by integrating deep features, achieving a precision of 0.91, recall of 0.87, and mAP@50 of 0.89, outperforming YOLOv10 and DETR. LW-DETR, a lightweight version of DETR, optimizes computational complexity but compromises detection accuracy, with precision of 0.88, recall of 0.84, and mAP@50 of 0.85, making it slightly less competitive than other models. In comparison, the proposed method outperforms all baselines, with a precision of 0.94, recall of 0.90, and mAP@50 and mAP@75 of 0.92 and 0.91, respectively. These results highlight the effectiveness and robustness of the proposed method in disease region localization and classification tasks.

From the perspective of mathematical properties and model architecture, the limitations of RetinaNet stem from its single-stage detector’s reliance on fixed anchor-based mechanisms, which struggle to handle complex disease morphologies and background interference [55]. DETR’s Transformer architecture captures global feature relationships through self-attention mechanisms, but its encoder–decoder architecture lacks progressive fine-grained feature optimization, which hampers its performance in small-object detection [57]. YOLOv10 introduces a lightweight network structure and multi-scale feature pyramid, enhancing the ability to model diverse disease features. However, its generalization ability under extreme lighting changes and complex disease patterns still falls short of Transformer-based models [58]. DETR v2 optimizes the Transformer’s feature extraction modules and improves loss functions and optimization strategies, enhancing its ability to model target regions and handle background noise [46]. In contrast, the proposed method leverages the endogenous diffusion sub-network to progressively model feature distributions, allowing the model to better preserve fine-grained disease features under lighting variations and complex backgrounds. Additionally, the design of the endogenous diffusion loss function integrates feature reconstruction, classification, and localization tasks, achieving multi-task joint optimization from a theoretical perspective. The diffusion model’s progressive denoising process dynamically adjusts weights, enabling the final feature distribution to effectively balance global semantic information and local detail features. This comprehensive analysis demonstrates the proposed method’s robustness and efficiency in complex scenarios, providing a novel solution for soybean disease detection tasks.

### 3.2. Results Analysis

The purpose of this experiment is to evaluate the performance of the proposed method in detecting different types of soybean diseases, validating the model’s suitability and generalizability for fine-grained classification tasks. The experiment focuses on five common soybean diseases: bacterial blight, Fusarium head blight, powdery mildew, root rot, and rust. The performance of the proposed method was analyzed in detail across these disease types, as shown in Table 3. This experiment not only quantifies the model’s ability to detect different diseases but also highlights the varying detection difficulties and the model’s performance across diverse disease characteristics.

The results indicate that the model achieves high performance across all disease types, with slight variations depending on the complexity and visual characteristics of each disease. For bacterial blight, the precision is 0.91, recall is 0.88, and AP@50 and AP@75 are 0.91 and 0.90, respectively. Despite the water-soaked spots of bacterial blight being easily confused in complex backgrounds, the model demonstrates strong detection accuracy by leveraging fine-grained feature extraction [59]. Fusarium head blight results are similar, with a precision of 0.92, recall of 0.89, and AP@50 and AP@75 at 0.92 and 0.91, respectively. The distinct red and purple streaks of Fusarium head blight allow the model to localize disease regions more accurately [60]. For powdery mildew, performance slightly improves with precision and recall at 0.93 and 0.91, and AP@50 and AP@75 at 0.92 and 0.91. The model’s robustness in handling this uniformly distributed powdery coverage on leaves is evident [61]. Root rot exhibits even better results, with precision at 0.95, recall at 0.92, and AP@50 and AP@75 at 0.93 and 0.92, showing the model’s strong capability to extract and classify localized features in root regions [62]. Finally, rust achieves the highest detection performance, with precision of 0.96, recall of 0.93, and AP@50 and AP@75 at 0.94 and 0.93, demonstrating the model’s superior ability to detect small, high-contrast features on leaves [63].

Analyzing these results from the perspective of mathematical characteristics and detection difficulties, the progressive modeling mechanism and the endogenous diffusion sub-network are key to the proposed method’s success. Diseases like bacterial blight and Fusarium head blight, characterized by complex and irregular morphological features, demand robust local feature extraction [64]. The proposed method achieves this through the endogenous diffusion sub-network, which progressively models features and integrates multi-scale representations, effectively separating disease regions from complex backgrounds. For powdery mildew and root rot, the relatively uniform features of powdery mildew and the localized prominence of root rot make these diseases more easily captured through the diffusion mechanism, which enhances fine-grained feature representation [61,62]. Consequently, the model performs better on these diseases than on bacterial blight and Fusarium head blight [60]. Rust, despite its small-sized features, benefits from high contrast and well-defined visual characteristics [63]. The diffusion mechanism’s stepwise denoising process preserves these salient features, and the dynamically weighted endogenous diffusion loss function further optimizes classification and localization accuracy, resulting in the highest performance for rust detection. From a theoretical perspective, the proposed method progressively optimizes feature distributions within the diffusion model, enabling the final features to balance global semantic information and fine-grained local feature representations. The endogenous diffusion loss function dynamically assigns weights to different disease types, allowing precise modeling of high-priority regions in scenarios with complex backgrounds and diverse disease characteristics. This design effectively mitigates challenges posed by uneven disease feature distributions, ensuring the model’s robustness and generalization across different disease types. In conclusion, the proposed method’s outstanding performance in fine-grained disease detection tasks underscores its broad applicability in complex agricultural scenarios, providing critical technological support for intelligent soybean disease detection.

### 3.3. Ablation Study on Different Attention Mechanisms

The purpose of this experiment was to evaluate the impact of different attention mechanisms on the performance of the object detection model in the soybean disease detection task. By comparing the model with no attention mechanism (“none” attention) and the endogenous diffusion attention mechanism, the aim was to quantify the contribution of the attention mechanism to the model’s precision, recall, accuracy, and mAP scores, thus validating the effectiveness of the endogenous diffusion attention mechanism in detecting disease regions. The experimental results show that the model with the endogenous diffusion attention mechanism significantly outperformed the model without any attention mechanism, particularly in terms of precision, recall, mAP@50, and mAP@75, demonstrating the robust performance of the attention mechanism in fine-grained feature modeling and complex backgrounds. The results are presented in Table 4.

In models without attention mechanisms, the feature extraction process relies solely on traditional convolutional networks, and the model processes input images in a more straightforward manner, lacking focused attention on different regions and details. These models typically depend on fixed feature extraction methods, which pose challenges in detecting complex backgrounds, lighting variations, or small targets. When dealing with fine-grained features of disease regions, the model often fails to effectively distinguish between background noise and the actual disease area, leading to lower detection precision and recall. In contrast, in the endogenous diffusion attention mechanism proposed in this paper, through two processes—forward diffusion and reverse generation—the model gradually optimizes the feature distribution, making feature extraction more refined and accurate. Forward diffusion simulates complex background and lighting interference by injecting noise, allowing the model to perform effective feature modeling in more complex environments. Reverse generation, through denoising operations, effectively suppresses background noise while highlighting the fine-grained features of the disease region. This multi-step feature modeling mechanism enables the model to gradually optimize feature expression from global to local, enhancing sensitivity to small disease regions. Compared to models without attention mechanisms, the endogenous diffusion attention mechanism dynamically weights features from different regions, allowing the model to more precisely model the fine-grained features of disease areas. For example, when dealing with diseases like rust and root rot, which have complex textures, the model can automatically focus on prominent features of the disease region, and through gradual denoising and optimization in different diffusion steps, it significantly improves detection accuracy. This attention mechanism allows the model to adapt to the diversity of disease features in different scenarios, maintaining high accuracy and robustness even in complex backgrounds. Further analysis shows that the endogenous diffusion attention mechanism optimizes feature distribution step by step, allowing the model to maintain high detection performance even when handling lighting changes, complex backgrounds, and large variations in target scales. In contrast, traditional models without attention mechanisms often experience significant performance degradation when dealing with these complex scenarios due to the lack of targeted optimization and adjustment of feature distribution. The advantage of the endogenous diffusion attention mechanism lies in its ability to autonomously focus on key regions of the image and still effectively extract disease features even in the presence of noise and interference, avoiding the recognition errors caused by insufficient feature extraction or excessive dependence on the background, which is common in traditional methods.

### 3.4. Discussion

In this study, we propose a deep learning-based soybean disease detection model and improve the model’s robustness and generalization ability through innovative data augmentation techniques. Existing research on soybean disease detection mainly relies on traditional machine learning methods and simple image-processing techniques, such as support vector machines (SVMs) and decision trees. These methods typically depend on handcrafted feature extraction and often perform poorly when faced with complex disease patterns [65,66]. While traditional methods, such as SVM, have been utilized for crop disease detection, they are limited by their inability to model complex, nonlinear relationships in high-dimensional data [67]. On the other hand, deep learning models, such as CNNs, automatically learn features and have demonstrated significant improvements in disease detection accuracy and efficiency [68]. The model proposed in this study is particularly effective in scenarios with limited data samples and class imbalance, which are common in agricultural datasets. Compared to traditional methods, it demonstrates stronger generalization ability, adapting well to complex disease patterns and diverse background conditions.

Furthermore, while previous studies have also applied deep learning methods for agricultural disease detection, most of them focus on recognizing a specific disease and use small datasets, limiting the generalizability of the models [69,70]. In contrast, this study broadens the scope of application by covering multiple types of soybean diseases and using large-scale datasets from various sources, which improves the model’s applicability in real-world scenarios. Previous models often struggled with generalization when exposed to new types of diseases or different environmental conditions. The larger, more diverse dataset used in this study ensures that the model can recognize a wide range of disease types, further enhancing its robustness in practical settings. This approach contrasts with studies that are often limited by smaller, specialized datasets, which may not be applicable in broader, real-world agricultural contexts. In comparison with other advanced models, such as RetinaNet, YOLOv10, and DETR, the proposed method stands out in terms of both performance and adaptability. RetinaNet’s reliance on fixed anchor boxes and a single-stage detection framework limits its ability to handle complex disease features and small objects in highly variable field conditions. YOLOv10, though fast and efficient, still faces challenges in real-world agricultural applications due to its inability to handle extreme lighting conditions and complex backgrounds effectively. DETR and DETR v2, based on Transformer architectures, offer improved performance by capturing global dependencies, but they still struggle with fine-grained details and small object detection in the presence of noisy backgrounds or subtle disease symptoms [71,72]. In contrast, our method’s use of an endogenous diffusion sub-network enhances feature extraction by progressively refining features through a noise-perturbation mechanism. This progressive feature modeling allows for better capture of fine-grained disease regions, even under challenging conditions, outperforming all baseline models in disease localization and classification tasks.

Moreover, the integration of a custom diffusion attention mechanism further boosts feature extraction accuracy and robustness. The method’s superior performance, as evidenced by its higher precision, recall, and mAP metrics compared to traditional attention mechanisms like self-attention and CBAM, highlights the strength of diffusion models in handling complex agricultural scenarios. Self-attention mechanisms, though effective in capturing long-range dependencies, tend to underperform when dealing with small objects or noisy data due to their computational complexity and inability to model fine-grained features effectively. In contrast, the proposed endogenous diffusion attention mechanism excels in small-object detection by progressively optimizing feature distributions, providing a clear advantage in scenarios with complex backgrounds and subtle disease signs. In summary, this study introduces significant advancements in deep learning methods for soybean disease detection. By leveraging innovative data augmentation techniques, a robust diffusion-based detection network, and a custom attention mechanism, our approach not only improves detection accuracy but also addresses challenges like class imbalance and data scarcity. The comparison with existing methods demonstrates that our model surpasses traditional machine learning and deep learning models in terms of generalization ability, robustness, and real-world applicability, offering a novel solution to the challenges of precision agriculture.

### 3.5. Application and Future Work

When the proposed model is applied to different field scenarios, several challenges may arise. First, variations in lighting conditions, background complexity, and disease characteristics across different field environments may significantly impact the generalization capability of the model. Ahmad et al. proposed a multi-dataset training scheme to evaluate the generalization ability of deep learning (DL) models under diverse environmental conditions. Experimental results indicated that DenseNet169 achieved a generalization accuracy of 81.60% when trained on RGBA images with background removal in the CD and S datasets, while training with a combination of PlantVillage and field data improved the accuracy to a range of 77.50% to 80.33% [73]. Askr et al. introduced a model utilizing ResNet50 for leaf feature extraction and employed the Copula entropy-optimized GWO algorithm for feature selection, enhancing feature quality and improving the search efficiency of GWO by 78.57%, thereby increasing the generalization ability for cotton disease detection [74]. Shoaib et al. proposed the EG-CNN model, which integrates omics data with hyperspectral images to predict plant disease types, achieving a test accuracy of 95.5% after training, thereby enhancing the generalization capability of plant disease detection [75]. Future research will build upon these findings to further explore the generalization of the proposed model. Furthermore, computational complexity and inference time are critical factors in practical applications. In scenarios involving mobile or edge computing devices, further optimization of the model structure may be required to achieve a balance between performance and efficiency. Ahmad et al. introduced an efficient CNN model incorporating statistical methods to address class imbalance while employing progressive transfer learning to improve convergence speed and mitigate overfitting and negative transfer. The model was tested on the PlantVillage and Korean pepper disease datasets, achieving accuracies of 99.69% and 99%, respectively [76]. Feng, Qian et al. optimized a lightweight CNN structure and deployed it on low-cost embedded devices. Experimental results demonstrated that MobileNetV2, Xception, and NasNetMobile achieved mean recognition accuracies of 0.978, 0.990, and 0.974, respectively, with on-site diagnostic accuracy exceeding 85%, significantly enhancing the precision and applicability of disease detection [77]. Rao, Deshen et al. proposed a pine wilt disease detection method based on deep learning and balance mixup. The NDVI threshold segmentation algorithm was utilized to automatically extract regions of interest (ROIs) from multispectral images, and the balance mixup was applied to generate augmented data, providing technical support for disease prevention and control [78]. Future studies will reference these approaches to further explore the balance between model performance and efficiency. Finally, substantial variations may exist in the types and manifestations of plant diseases across different regions, necessitating a high degree of adaptability in the model. Future research should focus on incorporating larger and more diverse field datasets while optimizing model lightweighting strategies. Quan, Siyu et al. proposed a lightweight CNN architecture for efficient crop disease diagnosis, making it suitable for low-computation devices [79]. Wang, YuYang et al. introduced the MixResCoAtNet model, based on the lightweight MixNet framework, to achieve accurate identification of rose diseases. This model outperformed traditional CNNs in accuracy while its lightweight design facilitated mobile deployment [80]. Chen, Yang et al. proposed the DFCANet model, which integrates dual-feature fusion with coordinate attention mechanisms to enable lightweight maize disease recognition, thereby improving both accuracy and computational efficiency [81]. Future research will build upon these approaches to further explore model lightweighting strategies.

## 4. Conclusions

The precise detection of soybean diseases is of great significance for the sustainable development of agricultural production. Traditional methods often fall short in terms of accuracy and robustness, especially when dealing with complex field scenarios. To address this issue, this paper proposes a diffusion-based object detection model, incorporating the endogenous diffusion sub-network and the endogenous diffusion loss function. These components enable precise modeling of complex backgrounds and diverse disease characteristics, both theoretically and practically. This research provides an innovative solution for intelligent disease detection in agriculture. The primary innovation of this work lies in the introduction of the diffusion mechanism for feature modeling. By progressively optimizing feature distributions, the model enhances its robustness in complex backgrounds and its sensitivity to disease regions. The endogenous diffusion sub-network employs forward diffusion and reverse generation processes to achieve multi-step feature modeling, effectively capturing fine-grained disease features. Additionally, the designed endogenous diffusion loss function integrates feature reconstruction, classification, and localization tasks, using dynamic weight adjustments to achieve multi-task joint optimization, thereby significantly improving detection performance. Based on this framework, the study conducts comprehensive comparative experiments with multiple classical detection models, as well as detailed evaluations of different attention mechanisms and disease types, systematically validating the proposed method’s effectiveness and applicability.

The experimental results demonstrate significant performance improvements achieved by the proposed method in soybean disease detection tasks. In comparative experiments with other models, the proposed method consistently outperformed all baselines, achieving a precision of 94%, recall of 90%, accuracy of 92%, and mAP@50 and mAP@75 of 92% and 91%, respectively. Compared with baseline models such as RetinaNet, DETR, YOLOv10, and DETR v2, the proposed method markedly improved the detection of small disease targets and complex backgrounds. In disease-specific detection experiments, the proposed method showed exceptional performance in rust detection, achieving a precision of 96% and a recall of 93%. For more complex diseases like bacterial blight and Fusarium head blight, the model also demonstrated strong feature extraction capabilities, with precision and mAP exceeding 90%. Moreover, in a comparative study of different attention mechanisms, the proposed endogenous diffusion attention exhibited unique advantages in feature extraction and disease detection, significantly outperforming standard self-attention and CBAM, achieving higher robustness and accuracy in complex scenarios. In conclusion, this work proposes a method with both theoretical innovation and practical significance for disease detection tasks. By introducing the diffusion mechanism and multi-task optimization strategies, the proposed method substantially enhances detection performance in complex agricultural scenarios.

In conclusion, this work proposes a method with both theoretical innovation and practical significance for disease detection tasks. By introducing the diffusion mechanism and multi-task optimization strategies, the proposed method substantially enhances detection performance in complex agricultural scenarios. However, future research can focus on improving the model’s adaptability to even more diverse and challenging field conditions, including variable lighting, different crop types, and extreme weather conditions. Additionally, integrating multi-modal data, such as environmental variables (temperature, humidity) and sensor-based data, could further enhance detection accuracy and generalization across diverse geographical locations. Furthermore, the efficiency of the model is a critical aspect for real-time deployment in agricultural settings, so further optimization of the model’s computational efficiency and reduction of inference time are important future directions. Finally, exploring the application of the diffusion mechanism in other agricultural tasks, such as pest detection or crop yield prediction, could open new avenues for the broader use of this innovative approach. The experimental results fully validate the effectiveness and applicability of this approach, providing critical technological support for the intelligent development of soybean disease detection and paving the way for broader applications of diffusion mechanisms in other domains.

## Figures and Tables

**Table 1:** Number of images for each disease.

Disease	Number of Images
Powdery mildew	803
Rust	1792
Root rot	1017
Fusarium head blight	1391
Bacterial blight	1598

**Table 2:** Model performance on disease detection tasks.

Model	Precision	Recall	Accuracy	mAP@50	mAP@75	mAP50-95
RetinaNet [43]	0.85	0.80	0.83	0.82	0.81	0.73
DETR [50]	0.87	0.83	0.85	0.85	0.83	0.76
YOLOv10 [44]	0.88	0.85	0.87	0.88	0.85	0.72
DETR v2 [51]	0.90	0.87	0.90	0.90	0.88	0.81
YOLOv11 [52]	0.89	0.86	0.87	0.87	0.86	0.68
D-FINE [53]	0.91	0.87	0.89	0.89	0.88	0.73
LW-DETR [54]	0.88	0.84	0.85	0.85	0.84	0.66
Proposed Method	0.94	0.90	0.92	0.92	0.91	0.85

**Table 3:** Detection results for different disease types.

Disease Type	Precision	Recall	Accuracy	AP@50	AP@75	AP50-95
Bacterial Blight	0.91	0.88	0.90	0.91	0.90	0.79
Fusarium Head Blight	0.92	0.89	0.91	0.92	0.91	0.72
Powdery Mildew	0.93	0.91	0.92	0.92	0.91	0.80
Root Rot	0.95	0.92	0.93	0.93	0.92	0.78
Rust	0.96	0.93	0.94	0.94	0.93	0.71

**Table 4:** Ablation study on attention mechanisms.

Model	Precision	Recall	Accuracy	mAP@50	mAP@75	mAP50-95
No Attention (baseline)	0.62	0.57	0.59	0.58	0.47	0.33
Proposed Method	0.94	0.90	0.92	0.92	0.91	0.85

## Data Availability

The data presented in this study are available upon request from the corresponding author.
